# ﻿Morpho-phylogenetic evidence reveals two new species and two new host records of *Diaporthe* (Diaporthales, Diaporthaceae) from *Phellodendron chinense* in Sichuan, China

**DOI:** 10.3897/mycokeys.123.162866

**Published:** 2025-10-15

**Authors:** Xinyue Li, Xiulan Xu, Shasha Xiang, Feng Liu, Feihu Wang, Xuejing Jiang, Yinggao Liu, Chunlin Yang

**Affiliations:** 1 College of Forestry, Sichuan Agricultural University, Chengdu 611130, China Sichuan Agricultural University Chengdu China; 2 Forest Ecology and Conservation in the Upper Reaches of the Yangtze River Key Laboratory of Sichuan Province, Sichuan Agricultural University, Chengdu 611130, China Chengdu Academy of Agricultural and Forestry Sciences Chengdu China; 3 Sichuan Mt. Emei Forest Ecosystem National Observation and Research Station, Sichuan Agricultural University, Chengdu 611130, China Sichuan Agricultural University Chengdu China; 4 Forestry Research Institute, Chengdu Academy of Agricultural and Forestry Sciences, Chengdu 611130, China Chengdu Academy of Agricultural and Forestry Sciences Chengdu China

**Keywords:** Ascomycota, coelomycetous asexual morph, phylogeny, sapbrobic fungi, Sordariomycetes, taxonomy

## Abstract

*Diaporthe* species are globally distributed with a wide variety of plant hosts inhabiting diverse ecological niches and functioning as endophytes, saprobes and pathogens. Numerous species in this genus are significant pathogens responsible for a wide range of diseases in both agricultural and forest plants. In this study, eight *Diaporthe* strains were isolated from branches of *Phellodendron chinense* collected in Sichuan Province, China. Using multi-locus phylogenetic analysis of five gene regions (ITS, *tef*1-α, *tub*2, *cal* and *his*3), along with morphological characterisation, two novel species — *D. phellodendri* and *D. leshanensis* — are introduced in the present study and two new host associations were recorded for *D. eucommiigena* and *D. litseae*. This study contributes to the growing understanding of *Diaporthe* diversity and host associations in China.

## ﻿Introduction

*Diaporthe* (syn. *Phomopsis*) is the type genus of the family Diaporthaceae, order Diaporthales (Nitschke 1867). Historically, *Diaporthe* (sexual morph) and *Phomopsis* (asexual morph) were considered as separate genera until the implementation of the “one fungus, one name” nomenclatural system, which resolved this taxonomic ambiguity by prioritising *Diaporthe*, the earlier-established name, as the accepted genus name (Rossman et al. 2015). Being a species-rich genus, *Diaporthe* currently comprises nearly 1,000 epithets listed in Species Fungorum (https://speciesfungorum.org/, accessed on 5 June 2025). Species of *Diaporthe* are globally distributed, functioning as pathogens, endophytes or saprobes across a wide range of hosts (Udayanga et al. 2014a; Dissanayake et al. 2020; Norphanphoun et al. 2022). In addition to this ecological versatility, *Diaporthe* species display distinct morphological characteristics in both their sexual and asexual stages. The sexual morph is characterised by aggregated ascomata with tapering necks, unitunicate 8-spored asci and ellipsoid to fusiform ascospores bearing prominent guttules. The asexual morph produces black, ostiolate pycnidia lined with cylindrical phialides that generate three types of hyaline, aseptate conidia: commonly observed alpha-conidia and beta-conidia and the rarely-encountered gamma-conidia (Gomes et al. 2013; Gao et al. 2017; Chaisiri et al. 2022; Liu et al. 2024; Li et al. 2025).

Morphological variation within *Diaporthe* is often insufficient for reliable species delimitation, as phenotypic traits can be influenced by host association and environmental factors, resulting in frequent misidentifications (Guo et al. 2020; Monkai et al. 2023; Aumentado and Balendres 2024a, b). To address this taxonomic challenge, multi-locus phylogenetic analyses, based on five gene loci (ITS, *tef*1-α, *tub*2, *cal* and *his*3), have substantially enhanced species resolution and clarified evolutionary relationships within the genus (Udayanga et al. 2012; Santos et al. 2017a). Although multi-locus phylogenetic analyses have improved species resolution in *Diaporthe*, challenges such as limited sampling and incomplete lineage sorting may still lead to inaccurate estimates of species diversity (Dissanayake et al. 2017b; Hilário et al. 2021a, b). To overcome these limitations, recent studies have applied GCPSR (genealogical concordance phylogenetic species recognition) and coalescent-based models, which have led to the synonymisation of previously over-split taxa and the reclassification of the genus into seven sections, including Betulicola, Crotalariae, Eres, Foeniculina, Psoraleae-pinnatae, Rudis and Sojae (Hilário et al. 2021a, b; Dissanayake et al. 2024).

Our ongoing research work has been dedicated to investigating fungi associated with woody medicinal plants in Sichuan, China, with particular attention to important native host species, such as *Phellodendron chinense*, *Eucommia ulmoides* and *Magnolia officinalis*. In recent investigations targeting *P. chinense* in Sichuan Province, several isolates belonging to the genus *Diaporthe* were obtained from branch tissues. Detailed morphological characterisation, combined with multilocus phylogenetic analysis, resulted in the identification of two novel species and two new host records. These findings expand the current taxonomic framework of *Diaporthe* and enhance our understanding of its host range and lineage diversification on woody medicinal plants.

## ﻿Materials and methods

### ﻿Sampling and fungal isolation

Samples from decaying branches of *Phellodendron chinense* were collected from the main planting regions in Leshan and Yibin, Sichuan Province, China, in May 2024. Samples were placed in sterile ziplock bags and transported to the laboratory for fungal isolation. Fungal isolation was carried out following the protocol described by Senanayake et al. (2020). All specimens were deposited in the Herbarium of Sichuan Agricultural University (SICAU), while living cultures were preserved in the Culture Collection of Sichuan Agricultural University (**SICAUCC**), Chengdu, China.

### ﻿Morphological characterisation

Morphological observations were conducted on the specimens collected from host materials. Macroscopic structures, including conidiomata, were examined under a dissecting microscope (NVT-GG; Shanghai Advanced Photoelectric Technology Co., Ltd., China) and photographed using a digital camera (VS-800C, Shenzhen Weishen Times Technology Co., Ltd., China). Microscopic features, including conidiophores and conidia, were observed using BX53 compound microscope (Olympus Corporation, Japan), equipped with an SD1600AC digital camera and CapStudio software (v. 3.8.10.0; Image Technology Company, Suzhou, China). Measurements of key features were taken using Tarosoft Image Framework v. 0.9.7 (Tarosoft (R), Nontha Buri, Thailand). Images were edited and compiled using Adobe Photoshop CC 2022 (Adobe Systems, San Jose, CA, USA).

### ﻿DNA extraction, PCR and sequencing

Genomic DNA was extracted from freshly growing mycelia on potato dextrose agar using the New Plant Genomic DNA Kit (Beijing Aidlab Biotechnologies Co., Ltd., Beijing, China), following the manufacturer’s protocol. Five gene regions, including the internal transcribed spacer (ITS), the translation elongation factor 1 alpha (*tef*1-α), the beta-tubulin (*tub*2), the calmodulin (*cal*) and the histone H3 (*his*3) were amplified using specific primer pairs. The corresponding primer sequences and thermal cycling conditions are provided in Table 1. PCR products were visualised on 2% agarose gels to verify successful amplification and subsequently sequenced bidirectionally using the same primers by Hangzhou Youkang Biotech Co., Ltd. (Chengdu, China). Consensus sequences were assembled using BioEdit v. 7.0.5.3 and submitted to GenBank. All obtained sequences were compared with those in GenBank using the BLASTn algorithm (http://www.ncbi.nlm.nih.gov/BLAST/) to determine the closest matches.

**Table 1.** Primers and PCR amplification procedures used in this study.

Gene	Primer	Primer sequence (5’-3’)	PCR condition	Reference
ITS	ITS5	GGAAGTAAAAGTCGTAACAAGG	94 °C for 3 min, 35 cycles of 94 °C for 30 s, 55 °C for 50 s and 72 °C for 1 min and 72 °C for 10 min	White et al. (1990)
	ITS4	TCCTCCGCTTATTGATATGC		
*tef*1-α	EF1-728F	CATCGAGAAGTTCGAGAAGG	94 °C for 3 min, 35 cycles of 94 °C for 30 s, 55 °C for 50 s and 72 °C for 1 min and 72 °C for 10 min	Carbone and Kohn (1999)
	EF1-986R	TACTTGAAGGAACCCTTACC		
*tub*2	Bt2a	GGTAACCAAATCGGTGCTGCTTTC	94 °C for 3 min, 35 cycles of 94 °C for 30 s, 51 °C for 50 s and 72 °C for 1 min and 72 °C for 10 min	Glass and Donaldson (1995), O’Donnell and Cigelnik (1997)
	Bt2b	ACCCTCAGTGTAGTGACCCTTGGC		
*cal*	CAL228F	GAGTTCAAGGAGGCCTTCTCCC	95 °C for 4 min, 35 cycles of 94 °C for 30 s, 60 °C for 50 s and 72 °C for 1 min and 72 °C for 10 min	Carbone and Kohn (1999)
	CAL737R	CATCTTTCTGGCCATCATGG		
*his*3	CYLH3F	AGGTCCACTGGTGGCAAG	96 °C for 5 min, 30 cycles of 96 °C for 30 s, 52 °C for 30 s and 72 °C for 1 min and 72 °C for 5 min	Glass and Donaldson (1995), Crous et al. (2004)
	x H3-1b	GCGGGCGAGCTGGATGTCCTT		

### ﻿Phylogenetic analyses

Sequences of *Diaporthe* species used for constructing the multigene dataset were downloaded from GenBank (Table 2). Multiple sequence alignments for each of the five gene regions (ITS, *tef*1-α, *tub*2, *cal* and *his*3) were performed separately using the online platform of MAFFT v. 7.490 (Katoh et al. 2019). Minor manual adjustments were subsequently done using BioEdit v. 7.0.5.3 (Hall 1999). The aligned gene regions were concatenated using PhyloSuite v. 1.2.3 (Zhang et al. 2020). Model selection was performed with the OFPT tool (Zeng et al. 2023). Phylogenetic trees were inferred using Maximum Likelihood (ML) in RAxML v. 8.2.12 and Bayesian Inference (BI) in MrBayes v. 3.2.7a, both run on the CIPRES Science Gateway (Stamatakis 2014). ML node support was evaluated with 1,000 bootstrap replicates, while BI posterior probabilities were estimated using MCMC sampling, which stopped automatically when the average standard deviation of split frequencies dropped below 0.01. Resulting phylogenetic trees were visualised and refined using ChiPlot (https://www.chiplot.online) (Xie et al. 2023) and Adobe Illustrator CS6 (Adobe Systems Inc., USA).

**Table 2.** GenBank accession numbers used in the phylogenetic analyses.

	Strains	GenBank accession number					Reference
		ITS	tef1-α	tub2	cal	his3	
Section Eres							
*Diaporthe alnicola*	CFCC 70997 ^T^	PQ636515	PQ635059	PQ635065	PQ635047	PQ635053	Li et al. (2025)
*D. alnicola*	CFCC 70998	PQ636516	PQ635060	PQ635066	PQ635048	PQ635054	Li et al. (2025)
*D. apiculata*	CGMCC3.17533 ^T^	KP267896	KP267970	KP293476	NA	NA	Gao et al. (2016)
*D. apiculata*	CFCC 53069	MK432652	MK578128	MK578055	MK442974	MK442999	Yang et al. (2021)
*D. apiculata*	LC3187	KP267866	KP267940	KP293446	NA	NA	Gao et al. (2016)
*D. azadirachtae*	TN 01	KC631323	NA	NA	NA	NA	Vedashree et al. (2015)
*D. charlesworthii*	BRIP 54884 m ^T^	KJ197288	KJ197250	KJ197268	NA	NA	Perera et al. (2018)
*D. citri*	CBS 135422 ^T^	KC843311	KC843071	KC843187	KC843157	MF418281	Udayanga et al. (2014b)
*D. citri*	AR4469	KC843321	KC843081	KC843197	KC843167	NA	Udayanga et al. (2014b)
*D. citrichinensis*	ZJUD34 ^T^	JQ954648	JQ954666	KJ490396	KC357494	KJ490516	Huang et al. (2015)
*D. citrichinensis*	ZJUD85	KJ490620	KJ490499	KJ490441	NA	KJ490562	Huang et al. (2015)
*D. collaria*	MFLUCC 17-2636 ^T^	MG806115	MG783040	MG783041	MG783042	NA	Perera et al. (2018)
*D. collaria*	SAUCC 194.12	MT822540	MT855854	MT855737	MT855625	MT855509	Sun et al. (2021)
*D. conica*	CFCC 52571 ^T^	MH121506	MH121548	MH121588	MH121428	MH121466	Yang et al. (2018a)
*D. conica*	CFCC 52572	MH121507	MH121549	MH121589	MH121429	MH121467	Yang et al. (2018a)
*D. corylopsidis*	CGMCC3.28211 ^T^	PQ319517	PQ336407	PQ336435	PQ336463	PQ336491	Zhang et al. (2025)
*D. corylopsidis*	SAUCC5501	PQ319518	PQ336408	PQ336436	PQ336464	PQ336492	Zhang et al. (2025)
*D. cryptostegiae*	CGMCC3.28204 ^T^	PQ319542	PQ336432	PQ336460	PQ336488	PQ336516	Zhang et al. (2025)
*D. cryptostegiae*	SAUCC0103	PQ319543	PQ336433	PQ336461	PQ336489	PQ336517	Zhang et al. (2025)
*D. eres*	AR5193 ^T^	KJ210529	KJ210550	KJ420799	KJ434999	KJ420850	Udayanga et al. (2014a)
*D. eres*	DLR12a	KJ210518	KJ210542	KJ420783	KJ434996	KJ420833	Udayanga et al. (2014a)
*D. eres*	LCM11401a	KJ210521	KJ210545	KJ420787	KJ435027	KJ420837	Udayanga et al. (2014a)
*D. fengmingensis*	CGMCC3.28225 ^T^	PQ319519	PQ336409	PQ336437	PQ336465	PQ336493	Zhang et al. (2025)
*D. fengmingensis*	SAUCC5330	PQ319520	PQ336410	PQ336438	PQ336466	PQ336494	Zhang et al. (2025)
*D. gardeniae*	CBS 288.56 ^T^	KC343113	KC343839	KC344081	KC343355	KC343597	Gomes et al. (2013)
*D. gardeniae*	step 1	KY797655	MF158048	MF158050	NA	MF158049	Li et al. (2023)
*D. grandiflori*	SAUCC194.84 ^T^	MT822612	MT855924	MT855809	MT855691	MT855580	Sun et al. (2021)
*D. hanceae*	CGMCC3.27974 ^T^	PQ319538	PQ336428	PQ336456	PQ336484	PQ336512	Zhang et al. (2025)
*D. hanceae*	SAUCC0164	PQ319539	PQ336429	PQ336457	PQ336485	PQ336513	Zhang et al. (2025)
*D. heterophyllae*	CBS 143769 ^T^	MG600222	MG600224	MG600226	MG600218	MG600220	Marin-Felix et al. (2019)
*D. irregularis*	CGMCC3.20092 ^T^	MT385951	MT424686	MT424706	MT424721	NA	Dissanayake et al. (2020)
*D. irregularis*	GZCC 19-0344	MT797179	MT793022	MT793033	MT786249	NA	Dissanayake et al. (2020)
*D. linzhiensis*	CFCC 71057 ^T^	PQ636519	PQ635063	PQ635069	PQ635051	PQ635057	Li et al. (2025)
*D. linzhiensis*	N266C	PQ636520	PQ635064	PQ635070	PQ635052	PQ635058	Li et al. (2025)
*D. litseae*	GUCC:24-0055 ^T^	PQ208460	PQ243584	NA	PQ213368	PQ213369	Sun et al. (2024)
*D. litseae*	GUCC:23-0022	PQ208459	PQ243583	NA	PQ213367	PQ213366	Sun et al. (2024)
***D. litseae***	**SICAUCC 25-0133**	PV741481	PV764957	PV764948	PV769972	PV759343	In this study
***D. litseae***	**SICAUCC 25-0134**	PV741482	PV764958	PV764949	PV769973	PV759344	In this study
*D. mianyangensis*	SICAUCC 23-0059 ^T^	PP060675	PP061149	PP061174	NA	NA	Wang et al. (2025)
*D. mianyangensis*	SICAUCC 23-0150	PP844878	PP850055	PP850062	NA	NA	Wang et al. (2025)
*D. oraccinii*	CGMCC3.17531 ^T^	KP267863	KP267937	KP293443	NA	KP293517	Gao et al. (2016)
*D. oraccinii*	LC3296	KP267884	KP267958	KP293464	NA	KP293536	Gao et al. (2016)
*D. penetriteum*	LC3353 ^T^	KP714505	KP714517	KP714529	NA	KP714493	Guo et al. (2020)
*D. penetriteum*	LC3215	KP267879	KP267953	KP293459	NA	KP293532	Guo et al. (2020)
*D. sennicola*	CFCC 51634 ^T^	KY203722	KY228883	KY228889	KY228873	KY228879	Yang et al. (2017)
*D. sennicola*	CFCC 51635	KY203723	KY228884	KY228890	KY228874	KY228880	Yang et al. (2017)
*D. shennongjiaensis*	CNUCC201905 ^T^	MN216229	MN224672	MN227012	MN224551	MN224560	Zhou et al. (2019)
*D. shennongjiaensis*	CNUCC 201906	MN216228	MN224673	MN227013	MN224552	MN224561	Zhou et al. (2019)
*D. subclavata*	ZJUD95 ^T^	KJ490630	KJ490509	KJ490451	NA	KJ490572	Sun et al. (2021)
*D. subclavata*	SAUCC3349	PQ319523	PQ336413	PQ336441	PQ336469	PQ336497	Zhang et al. (2025)
*D. virgiliae*	CMW 40755 ^T^	KP247573	NA	KP247582	NA	NA	Machingambi et al. (2015)
*D. virgiliae*	CMW 40748	KP247566	NA	KP247575	NA	NA	Machingambi et al. (2015)
*D. zaofenghuang*	CGMCC3.20271 ^T^	MW477883	MW480871	MW480875	MW480867	MW480863	Wang et al. (2021)
*D. zaofenghuang*	TZFH3	MW477884	MW480872	MW480876	MW480868	MW480864	Wang et al. (2021)
Section Sojae							
*Diaporthe acaciarum*	CBS 138862 ^T^	KP004460	NA	KP004509	NA	KP004504	Crous et al. (2014)
*D. alpiniae*	CGMCC3.28221 ^T^	PQ321210	PQ336519	PQ336537	PQ336555	PQ336573	Zhang et al. (2025)
*D. alpiniae*	SAUCC3248	PQ321211	PQ336520	PQ336538	PQ336556	PQ336574	Zhang et al. (2025)
*D. amaranthophila*	MAFF 246900 ^T^	LC459575	LC459577	LC459579	LC459583	LC459581	Rossman et al. (2015)
*D. amaranthophila*	MAFF 246901	LC459576	LC459578	LC459580	LC459584	LC459582	Rossman et al. (2015)
*D. ambigua*	CBS 114015 ^T^	MH862953	KC343736	KC343978	KC343252	KC343494	Vu et al. (2019)
*D. ambigua*	CBS 117167	KC343011	KC343737	KC343979	KC343253	KC343495	Vu et al. (2019)
*D. angelicae*	CBS 111592 ^T^	KC343027	KC343753	KC343995	KC343269	KC343511	Gomes et al. (2013)
*D. angelicae*	CBS 100871	KC343025	KC343751	KC343993	KC343267	KC343509	Gomes et al. (2013)
*D. arctii*	CBS 139280 ^T^	KJ590736	KJ590776	KJ610891	KJ612133	KJ659218	Udayanga et al. (2015)
*D. arezzoensis*	MFLU 19-2880 ^T^	MT185503	MT454019	MT454055	NA	NA	Li et al. (2020)
*D. batatas*	CBS 122.21 ^T^	KC343040	KC343766	KC344008	KC343282	KC343524	Udayanga et al. (2015)
*D. beilharziae*	BRIP 54792 ^T^	JX862529	JX862535	KF170921	NA	NA	Tan et al. (2013)
*D. betae*	HMPHU 3001 ^T^	MW882216	MW882222	MW882228	MW882219	MW882225	Shao et al. (2025)
*D. betae*	HUMCC 3268	MW882217	MW882223	MW882229	MW882220	MW882226	Shao et al. (2025)
*D. biguttulata*	CFCC 52584 ^T^	MH121519	MH121561	MH121598	MH121437	MH121477	Gao et al. (2015)
*D. biguttulata*	CFCC 52585	MH121520	MH121562	MH121599	MH121438	MH121478	Gao et al. (2015)
*D. brasiliensis*	CBS 133183 ^T^	KC343042	KC343768	KC344010	KC343284	KC343526	Dissanayake et al. (2017b)
*D. brasiliensis*	LGMF926	KC343043	KC343769	KC344011	KC343285	KC343527	Dissanayake et al. (2017b)
*D. breyniae*	CBS 148910 ^T^	ON400846	ON409188	ON409186	ON409189	ON409187	Matio Kemkuignou et al. (2022)
*D. caatingaensis*	URM7485	KY085927	KY115604	KY115601	KY115598	KY115605	Crous et al. (2016)
*D. caatingaensis*	URM7484	KY085928	NA	KY115602	KY115599	KY115606	Crous et al. (2016)
*D. caryae*	CFCC 52563 ^T^	MH121498	MH121540	MH121580	MH121422	MH121458	Yang et al. (2018b)
*D. caryae*	CFCC 52564	MH121499	MH121541	MH121581	MH121423	MH121459	Yang et al. (2018b)
*D. chiangraiensis*	MFLUCC 17-1669 ^T^	MF190119	MF377598	NA	NA	NA	Senanayake et al. (2017)
*D. chiangraiensis*	MFLUCC 17-1670	MF190118	MF377599	NA	NA	NA	Senanayake et al. (2017)
*D. chimonanthi*	SCHM 3614 ^T^	AY622993	NA	NA	NA	NA	Chang et al. (2005)
*D. chimonanthi*	SCHM 3603	AY620820	NA	NA	NA	NA	Chang et al. (2005)
*D. cichorii*	MFLUCC 17-1023 ^T^	KY964220	KY964176	KY964104	KY964133	NA	Dissanayake et al. (2017b)
*D. cinnamomi*	CFCC 52569 ^T^	MH121504	MH121546	MH121586	NA	MH121464	Zhu et al. (2023)
*D. cinnamomi*	CFCC 52570	MH121505	MH121547	MH121587	NA	MH121465	Zhu et al. (2023)
*D. citriasiana*	ZJUD30 ^T^	JQ954645	JQ954663	KC357459	KC357491	NA	Huang et al. (2013)
*D. citriasiana*	ZJUD81	KJ490616	KJ490495	KJ490437	NA	KJ490558	Huang et al. (2015)
*D. convolvuli*	CBS 124654 ^T^	KC343054	KC343780	KC344022	KC343296	KC343538	Dissanayake et al. (2017b)
*D. convolvuli*	FAU649	KJ590721	KJ590765	NA	KJ612130	KJ659210	Udayanga et al. (2015)
*D. coracoralinae*	URM 8912 ^T^	NR_198716	PP430449	PP402241	PP408214	PP421133	Ferro et al. (2024)
*D. cucurbitae*	DAOM 42078 ^T^	KM453210	KM453211	KP118848	NA	KM453212	Udayanga et al. (2015)
*D. cucurbitae*	CBS 136.25	KC343031	KC343757	KC343999	KC343273	KC343515	Gomes et al. (2013)
*D. cuppatea*	CBS 117499 ^T^	KC343057	KC343783	KC344025	KC343299	KC343541	Van et al. (2006)
*D. cyatheae*	YMJNA1364 ^T^	JX570889	KC465406	KC465403	KC465410	NA	Fu et al. (2013)
*D. desmotis*	CGMCC3.28203 ^T^	PQ321216	PQ336525	PQ336543	PQ336561	PQ336579	Zhang et al. (2025)
*D. desmotis*	SAUCC1130	PQ321217	PQ336526	PQ336544	PQ336562	PQ336580	Zhang et al. (2025)
*D. discoidispora*	ZJUD89 ^T^	KJ490624	KJ490503	KJ490445	NA	KJ490566	Huang et al. (2015)
*D. discoidispora*	ZJUD87	KJ490622	KJ490501	KJ490443	NA	KJ490564	Huang et al. (2015)
*D. eleutharrhenae*	1 ^T^	OK017069	OK017070	OK017071	NA	NA	Song et al. (2022)
*D. eleutharrhenae*	2	OK648457	OK648458	OK648459	NA	NA	Song et al. (2022)
*D. ervatamiae*	HKAS 138717 ^T^	PQ637066	NA	NA	NA	NA	Sun et al. (2025)
*D. eucommiigena*	GUCC 420.19	OP581224	OP688529	OP688554	NA	NA	Wang et al. (2022)
*D. eucommiigena*	GUCC 420.9^T^	OP581223	OP688528	OP688553	NA	NA	Wang et al. (2022)
***D. eucommiigena***	**SICAUCC 25-0131**	PV741477	PV764953	PV764944	NA	PV759339	In this study
***D. eucommiigena***	**SICAUCC 25-0132**	PV741478	PV764954	PV764945	NA	PV759340	In this study
*D. fici-macrocarpae*	SAUCC0412 ^T^	PQ321225	PQ336534	PQ336552	PQ336570	PQ336588	Zhang et al. (2025)
*D. fici-macrocarpae*	SAUCC0141	PQ321226	PQ336535	PQ336553	PQ336571	PQ336589	Zhang et al. (2025)
*D. fici-septicae*	NCYUCC 19-0108 ^T^	MW114349	MW192212	MW148269	NA	NA	Tennakoon et al. (2021)
*D. fici-septicae*	MFLU 20-20178	MW114348	MW192211	MW148268	NA	NA	Tennakoon et al. (2021)
*D. foliorum*	CMRP 1321 ^T^	MT576688	MT584310	MT584327	MT584341	MT584338	dos Santos et al. (2021)
*D. foliorum*	CMRP 1330	MT576671	MT584309	MT584328	MT584342	MT584340	dos Santos et al. (2021)
*D. ganjae*	CBS 180.91 ^T^	KC343112	KC343838	KC344080	KC343354	KC343596	Gomes et al. (2013)
*D. ganjae*	CGMCC3.17536	KP267854	KP267928	KP293434	NA	KP293508	Gao et al. (2016)
*D. goulteri*	BRIP 55657a ^T^	KJ197290	KJ197252	KJ197270	NA	NA	Thompson et al. (2015)
*D. guangzhouensis*	CFCC 58151 ^T^	PP864725	PP938601	PP938605	PP938609	PP938613	Zhu et al. (2024)
*D. gulyae*	BRIP 54025 ^T^	JF431299	JN645803	NA	NA	NA	Thompson et al. (2011)
*D. gulyae*	BRIP 53158	JF431284	JN645799	NA	NA	NA	Thompson et al. (2011)
*D. guttulata*	CGMCC3.20100 ^T^	MT385950	MT424685	MT424705	MW022470	MW022491	Dissanayake et al. (2020)
*D. guttulata*	GZCC 19-0371	MT797178	MT793021	MT793032	MW022471	MW022492	Dissanayake et al. (2020)
*D. helianthi*	CBS 592.81 ^T^	KC343115	KC343841	KC344083	KC343357	KC343599	Gomes et al. (2013)
*D. helianthi*	CBS 344.94	KC343114	KC343840	KC344082	KC343356	KC343598	Gomes et al. (2013)
*D. hordei*	CBS 481.92 ^T^	KC343120	KC343846	KC344088	KC343362	KC343604	Gomes et al. (2013)
*D. infecunda*	CBS 133812 ^T^	KC343126	KC343852	KC344094	KC343368	KC343610	Gomes et al. (2013)
*D. infertilis*	CBS 230.52 ^T^	KC343052	KC343778	KC344020	KC343294	KC343536	Guarnaccia et al. (2017)
*D. infertilis*	CPC 20322	KC343053	KC343779	KC344021	KC343295	KC343537	Guarnaccia et al. (2017)
*D. juglandige*	GUCC 422.16 ^T^	OP581229	OP688534	OP688559	NA	NA	Wang et al. (2022)
*D. juglandige*	GUCC 422.161	OP581230	OP688535	OP688560	NA	NA	Wang et al. (2022)
*D. kunmingensis*	HKAS 136905 ^T^	PQ637068	NA	NA	NA	NA	Sun et al. (2025)
*D. kyushuensis*	STENAU2675 ^T^	AF230749	NA	NA	NA	NA	Mostert et al. (2001)
*D. kyushuensis*	chNADNA1	AB302250	NA	NA	NA	NA	Mostert et al. (2001)
*D. leucospermi*	CBS 111980 ^T^	NA	KY435632	KY435673	KY435663	KY435653	Hilário et al. (2020)
*D. leucospermi*	CAA762	MK792290	MK828063	MK837914	MK883822	MK871432	Hilário et al. (2020)
***D. leshanensis***	**SICAUCC 25-0129**	PV741475	PV764951	PV764942	PV769970	PV759337	In this study
***D. leshanensis***	**SICAUCC 25-0130 ^T^**	PV741476	PV764952	PV764943	PV769971	PV759338	In this study
*D. longicolla*	FAU599 ^T^	KJ590728	KJ590767	KJ610883	KJ612124	KJ659188	Udayanga et al. (2015)
*D. longicolla*	FAU644	KJ590730	KJ590769	KJ610885	KJ612126	KJ659190	Udayanga et al. (2015)
*D. longispora*	CBS 194.36 ^T^	KC343135	KC343861	KC344103	KC343377	KC343619	Gomes et al. (2013)
*D. lusitanicae*	CBS 123213 ^T^	KC343137	KC343863	KC344105	KC343379	KC343621	Gomes et al. (2013)
*D. lusitanicae*	CBS 123212	KC343136	KC343862	KC344104	KC343378	KC343620	Gomes et al. (2013)
*D. machilii*	SAUCC194.111 ^T^	MT822639	MT855951	MT855836	MT855718	MT855606	Huang et al. (2021)
*D. mayteni*	CBS 133185 ^T^	KC343139	KC343865	KC344107	KC343381	KC343623	Gomes et al. (2013)
*D. megalospora*	CBS 143.27 ^T^	KC343140	KC343866	KC344108	KC343382	KC343624	Gomes et al. (2013)
*D. melongenae*	MBELPIC61.1 ^T^	OQ123525	OR099712	OR099714	NA	NA	Aumentado et al. (2024a)
*D. melongenicola*	CGMCC3.27978 ^T^	PQ321221	PQ336530	PQ336548	PQ336566	PQ336584	Zhang et al. (2025)
*D. melongenicola*	SAUCC0472	PQ321222	PQ336531	PQ336549	PQ336567	PQ336585	Zhang et al. (2025)
*D. melonis*	CBS 507.78 ^T^	KC343142	KC343868	KC344110	KC343384	KC343626	Gomes et al. (2013)
*D. melonis*	FAU640	KJ590702	KJ590741	KJ610858	KJ612099	KJ659184	Udayanga et al. (2015)
*D. middletonii*	BRIP 54884e ^T^	KJ197286	KJ197248	KJ197266	NA	NA	Thompson et al. (2015)
*D. middletonii*	BRIP 57329	KJ197285	KJ197247	KJ197265	NA	NA	Thompson et al. (2015)
*D. minusculata*	CGMCC3.20098 ^T^	MT385957	MT424692	MT424712	MW022475	MW022499	Dissanayake et al. (2020)
*D. minusculata*	GZCC 19-0345	MT797184	MT793027	MT793038	MW022476	MW022500	Dissanayake et al. (2020)
*D. monetii*	MF Ha18-048 ^T^	MW008493	MW008515	MW008504	MZ671938	MZ671964	Gomzhina et al. (2022)
*D. monetii*	MF Ha18-049	MW008494	MW008516	MW008505	MZ671939	MZ671965	Gomzhina et al. (2022)
*D. morindendophytica*	ZHKUCC 22-0069 ^T^	ON322897	ON315053	ON315087	NA	ON315027	Luo et al. (2022)
*D. morindendophytica*	ZHKUCC 22-0070	ON322898	ON315054	ON315088	NA	ON315028	Luo et al. (2022)
*D. myracrodruonis*	URM 7972 ^T^	MK205289	MK213408	MK205291	MK205290	NA	da Silva et al. (2019)
*D. neoarctii*	CBS 109490 ^T^	KC343145	KC343871	KC344113	KC343387	KC343629	Gomes et al. (2013)
*D. novem*	CBS 127270 ^T^	KC343156	KC343882	KC344124	KC343398	KC343640	Gomes et al. (2013)
*D. novem*	CBS 127271	KC343157	KC343883	KC344125	KC343399	KC343641	Gomes et al. (2013)
*D. orixae*	KUNCC 21-10714 ^T^	OK283041	NA	NA	OK484485	OK484486	Lu et al. (2022)
*D. orixae*	GZCC 21-1085	OL889852	OL944724	OL944726	NA	NA	Lu et al. (2022)
*D. ovalispora*	CGMCC3.17256 ^T^	KJ490628	KJ490507	KJ490449	NA	KJ490570	Huang et al. (2015)
*D. oxe*	CBS 133186 ^T^	KC343164	KC343890	KC344132	KC343406	KC343648	Gomes et al. (2013)
*D. oxe*	CBS 133187	KC343165	KC343891	KC344133	KC343407	KC343649	Gomes et al. (2013)
*D. pachirae*	CDA 728 ^T^	MG559537	MG559539	MG559541	MG559535	NA	Milagres et al. (2018)
*D. pachirae*	CDA 730	MG559538	MG559540	MG559542	MG559536	NA	Milagres et al. (2018)
*D. paranensis*	CBS 133184 ^T^	KC343171	KC343897	KC344139	KC343413	KC343655	Gomes et al. (2013)
*D. paranensis*	LMICRO417	KY461115	KY461116	NA	NA	NA	dos Santos et al. (2021)
*D. passiflorae*	CBS 132527 ^T^	JX069860	KY435633	KY435674	KY435664	KY435654	Crous et al. (2012)
*D. passiflorae*	CAA734	KY435638	KY435627	KY435668	KY435658	KY435648	Santos et al. (2017b)
*D. passiflorae*	CAA953	MN190308	MT309430	MT309456	MT309447	MT309439	Santos et al. (2017b)
*D. pedratalhadensis*	URM8304 ^T^	PP192073	PP430438	PP402232	PP408216	PP421129	Ferro et al. (2024)
*D. phaseolorum*	AR4203 ^T^	KJ590738	KJ590739	KJ610893	KJ612135	KJ659220	Gomes et al. (2013)
***D. phellodendri***	**SICAUCC 23-0174 ^T^**	PV741479	PV764955	PV764946	NA	PV759341	In this study
***D. phellodendri***	**SICAUCC 23-0175**	PV741480	PV764956	PV764947	NA	PV759342	In this study
*D. pseudofoliicola*	HNCM045 ^T^	OR647680	OR671940	OR671948	NA	OR671932	Liu et al. (2024)
*D. pygmaeae*	CDP 1370 ^T^	PP577992	PP579317	PP579332	PP579348	NA	Pereira et al. (2024)
*D. quercicola*	CSUFTCC104 ^T^	ON076567	ON081659	NA	ON081670	ON081667	Cao et al. (2022)
*D. quercicola*	CSUFTCC105	ON076568	ON081660	NA	ON081671	ON081668	Cao et al. (2022)
*D. racemosae*	CBS 143770 ^T^	MG600223	MG600225	MG600227	MG600219	MG600221	Marin-Felix et al. (2019)
*D. raonikayaporum*	CBS 133182 ^T^	KC343188	KC343914	KC344156	KC343430	KC343672	Gomes et al. (2013)
*D. raonikayaporum*	MFLUCC 14-1133	KU712448	KU749368	KU743987	KU749355	NA	Doilom et al. (2017)
*D. rosae*	MFLUCC 17-2658 ^T^	MG828894	NA	MG843878	MG829273	NA	Wanasinghe et al. (2018)
*D. rosae*	MFLUCC 17-2574	MG906793	MG968954	MG968952	NA	NA	Wanasinghe et al. (2018)
*D. rosiphthora*	COAD 2913 ^T^	MT311197	MT313693	NA	MT313691	NA	Pereira et al. (2021)
*D. sackstonii*	BRIP 54669b ^T^	KJ197287	KJ197249	KJ197267	NA	NA	Thompson et al. (2015)
*D. schini*	CBS 133181 ^T^	KC343191	KC343917	KC344159	KC343433	KC343675	Gomes et al. (2013)
*D. schini*	LGMF 910	KC343192	KC343918	KC344160	KC343434	KC343676	Gomes et al. (2013)
*D. schoeni*	MFLU 15-1279 ^T^	KY964226	KY964182	KY964109	KY964139	NA	Dissanayake et al. (2017a)
*D. schoeni*	MFLU 15-2609	KY964229	KY964185	KY964112	KY964141	NA	Dissanayake et al. (2017a)
*D. sclerotioides*	CBS 296.67 ^T^	MH858974	KC343919	KC344161	KC343435	KC343677	Gomes et al. (2013)
*D. sclerotioides*	CBS 710.76	KC343194	KC343920	KC344162	KC343436	KC343678	Gomes et al. (2013)
*D. serafiniae*	BRIP 55665a ^T^	KJ197274	KJ197236	KJ197254	NA	NA	Thompson et al. (2015)
*D. serafiniae*	BRIP 54136	KJ197273	KJ197235	KJ197253	NA	NA	Thompson et al. (2015)
*D. siamensis*	MFLUCC 10NA0573a ^T^	JQ619879	JX275393	JX275429	JX197423	NA	Udayanga et al. (2012)
*D. siamensis*	MFLUCC 12-0300	KT459417	KT459451	KT459435	KT459467	NA	Doilom et al. (2017)
*D. sinoadinae*	CGMCC3.27970 ^T^	PQ321214	PQ336523	PQ336541	PQ336559	PQ336577	Zhang et al. (2025)
*D. sinoadinae*	SAUCC5606	PQ321215	PQ336524	PQ336542	PQ336560	PQ336578	Zhang et al. (2025)
*D. sojae*	FAU635 ^T^	KJ590719	KJ590762	KJ610875	KJ612116	KJ659208	Udayanga et al. (2015)
*D. sojae*	AR3602	KJ590714	KJ590757	KJ610870	KJ612111	KJ659203	Udayanga et al. (2015)
*D. sojae*	CBS 116019	KC343175	KC343901	KC344143	KC343417	KC343659	Gomes et al. (2013)
*D. solani*-*melongenae*	MCCNAMNH 2729 ^T^	OQ123551	OR943642	OR943679	NA	NA	Aumentado et al. (2024b)
*D. stewartii*	CBS 193.36	MH867279	GQ250324	JX275421	JX197415	NA	Santos et al. (2010)
*D. stewartii*	MN1	KX668416	KX852355	NA	NA	NA	Olson et al. (2017)
*D. submersa*	CGMCC3.24297 ^T^	OP056717	OP150556	OP150633	OP150710	OP150786	Dissanayake et al. (2024)
*D. submersa*	GZCC 22-0007	OP056718	OP150557	OP150634	OP150711	OP150787	Dissanayake et al. (2024)
*D. subordiria*	CBS 464.90 ^T^	KC343214	KC343940	KC344182	KC343456	KC343698	Gomes et al. (2013)
*D. subordiria*	CBS 101711	KC343213	KC343939	KC344181	KC343455	KC343697	Gomes et al. (2013)
*D. talong*	MCCNAMNH 2727 ^T^	OQ123540	OR943636	OR943673	NA	NA	Aumentado et al. (2024a)
*D. tarchonanthi*	CBS 146073 ^T^	MT223794	NA	MT223733	NA	MT223759	Gomes et al. (2013)
*D. tecomae*	CBS 100547 ^T^	KC343215	KC343941	KC344183	KC343457	KC343699	Doilom et al. (2017)
*D. tectonendophytica*	MFLUCC 13-0471 ^T^	KU712439	KU749367	KU743986	KU749354	NA	Doilom et al. (2017)
*D. tectonendophytica*	LC8115	KY491550	KY491560	KY491570	NA	NA	Gao et al. (2017)
*D. terebinthifolii*	CBS 133180 ^T^	KC343216	KC343942	KC344184	KC343458	KC343700	Gomes et al. (2013)
*D. terebinthifolii*	LGMF907	KC343217	KC343943	KC344185	KC343459	KC343701	Gomes et al. (2013)
*D. thunbergiicola*	MFLUCC 12-0033 ^T^	KP715097	KP715098	NA	NA	NA	Liu et al. (2015)
*D. tulliensis*	BRIP 62248a ^T^	KR936130	KR936133	KR936132	NA	NA	Crous et al. (2015)
*D. ueckerae*	FAU656 ^T^	KJ590726	KJ590747	KJ610881	KJ612122	KJ659215	Huang et al. (2015)
*D. ueckerae*	BRIP 54736j	KJ197282	KJ197244	KJ197262	NA	NA	Thompson et al. (2015)
*D. unshiuensis*	ZJUD50 ^T^	KJ490585	KJ490464	KJ490406	NA	KJ490527	Huang et al. (2015)
*D. unshiuensis*	ZJUD52	KJ490587	KJ490466	KJ490408	NA	KJ490529	Huang et al. (2015)
*D. vangoghii*	MF Ha18-045 ^T^	MW008491	MW008513	MW008502	MZ671936	MZ671962	Gomzhina et al. (2022)
*D. vangoghii*	MF Ha18-046	MW008492	MW008514	MW008503	MZ671937	MZ671963	Gomzhina et al. (2022)
*D. vargemgrandensis*	URM8784 ^T^	PP192069	PP430456	PP421092	PP421068	PP421135	Ferro et al. (2024)
*D. vexans*	CBS 127.14	KC343229	KC343955	KC344197	KC343471	KC343713	Crous et al. (2015)
*D. vexans*	FAU597	KJ590734	KJ590774	KJ610889	KJ612131	KJ659216	Udayanga et al. (2015)
*D. vochysiae*	LGMF1583 ^T^	MG976391	MK007526	MK007527	MK007528	MK033323	Noriler et al. (2019)
*D. yunnanensis*	CGMCC3.18289 ^T^	KX986796	KX999188	KX999228	KX999290	KX999267	Gao et al. (2017)
*D. yunnanensis*	LC8107	KY491542	KY491552	KY491562	KY491572	NA	Gao et al. (2017)
*Diaporthella corylina*	CBS 121124	KC343004	KC343730	KC343972	KC343246	KC343488	Gomes et al. (2013)

Note: The newly-generated sequences are indicated in bold. Ex-type or ex-epitype strains are marked with “T”. “NA” means sequence is unavailable.

### ﻿Genealogical concordance phylogenetic species recognition analysis

The Genealogical Concordance Phylogenetic Species Recognition (GCPSR) approach was applied to assess potential recombination amongst phylogenetically closely-related taxa, based on the multi-locus sequence data (Taylor et al. 2000). Five gene regions (ITS, *tef*1-α, *tub*2, *cal* and *his*3) were aligned and concatenated into a single dataset. The concatenated alignment was analysed in SplitsTree v.4.17.1 (Huson and Bryant 2006) using the Pairwise Homoplasy Index (PHI, *Φw*) test to detect signals of recombination. Significant recombination was inferred when *Φw* < 0.05. The relationships amongst the closely-related taxa were further visualised by constructing a split network using the LogDet transformation and Splits options.

## ﻿Results

### ﻿Phylogenetic analysis

For all eight isolates, BLASTn results for ITS, *tef*1-α, *tub*2, *cal* and *his*3 consistently matched *Diaporthe*, confirming placement at the generic level. To confirm the phylogenetic position, representative sequences of *Diaporthe* were downloaded from GenBank and incorporated into a combined dataset (ITS, *tef*1-α, *tub*2, *cal* and *his*3), which placed our isolates within the genus *Diaporthe*, distributed across two major sections corresponding to Section Sojae and Section Eres (Dissanayake et al. 2024). Finally, a combined five-gene dataset comprising 229 in-group taxa in *Diaporthe*, belonging to Section Sojae and Section Eres and one out-group taxon (*D. corylina*, CBS 121124) was used to construct the phylogenetic tree. The alignment contained a total of 3,884 characters (ITS: 1–663, *tef*1-α: 664–1,533, *tub*2: 1,534–2,569, *his*3: 2,570–3,201, *cal*: 3,202–3,884) after alignment. Single-gene analyses were conducted and they resulted in similar tree topologies between the ML and BI methods, ensuring the consistent comparisons of clade stability. The best-scoring RAxML tree with a final likelihood value of –58,926.815664 is presented. The matrix had 2,642 distinct alignment patterns, with 46.91% of undetermined characters or gaps. Estimated base frequencies were as follows: A = 0.214476, C = 0.325660, G = 0.238024, T = 0.221840; substitution rates AC = 1.190748, AG = 3.397866, AT = 1.269937, CG = 0.994576, CT = 4.742139, GT = 1.000000. Bayesian analyses reached convergence, with a final average standard deviation of split frequencies of 0.009999.

**Figure 1.**
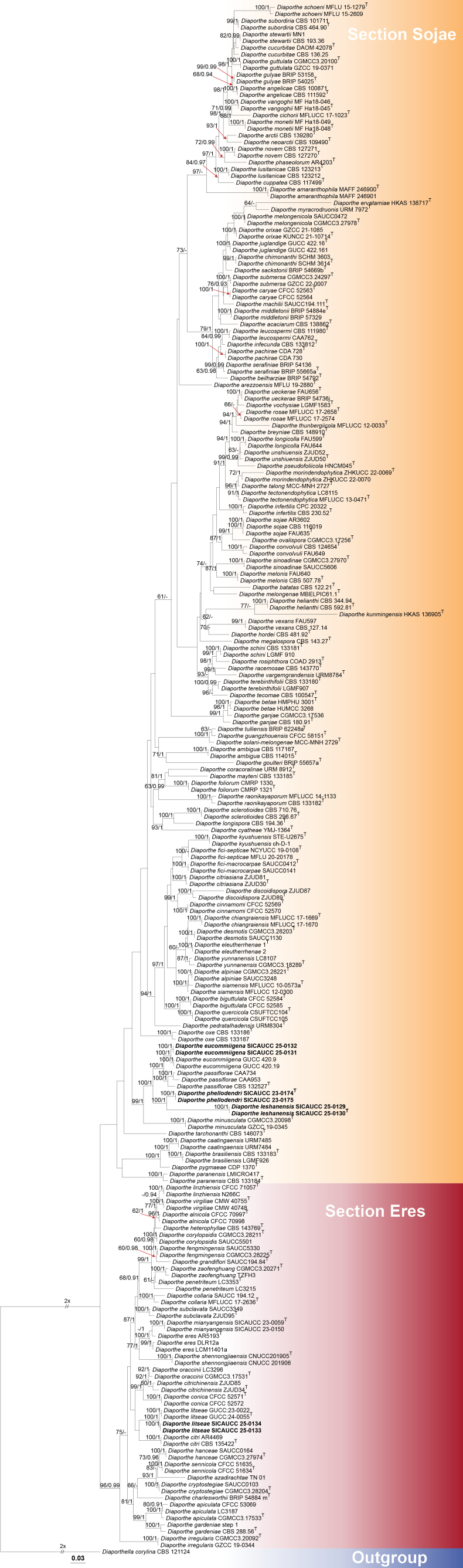
Maximum Likelihood phylogenetic tree, based on concatenated sequences of the ITS, *tef*1-α, *tub*2, *cal* and *his*3 gene regions from *Diaporthe* species. Bootstrap support values ≥ 60% (left) and Bayesian posterior probabilities ≥ 0.90 (right) are shown at the nodes. Isolates from the present study are indicated in bold. Ex-type or ex-epitype strains are marked with “T”.

**Figure 2.**
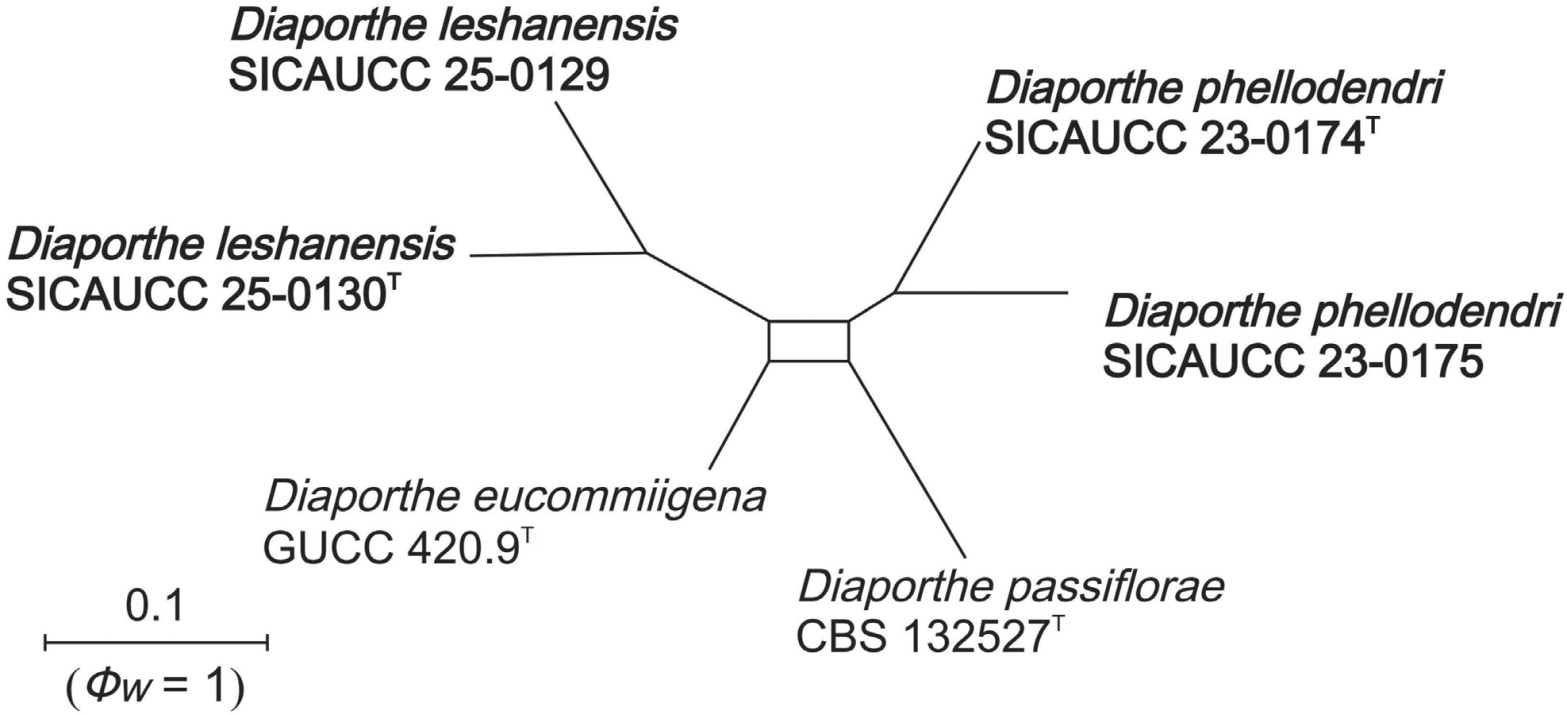
The splits graph from the pairwise homoplasy index (PHI) test generated from the concatenated gene set of ITS, *tef*1-α, *tub*2, *cal* and *his*3 sequence data of closely-related species of *Diaporthe phellodendri* and *D. leshanensis* using both LogDet transformation and splits decomposition. PHI test results (*Фw*) < 0.05 indicate significant recombination within the dataset. The strains determined in this study are in bold. Ex-type or ex-epitype strains are marked with “T”.

Based on the phylogenetic tree, six isolates are phylogenetically grouped within Section Sojae (Fig. 1). Two new species identified as *Diaporthe phellodendri* and *D. leshanensis* are clustered in two independent clades within this section. The isolates SICAUCC 25-0131 and SICAUCC 25-0132 clustered with strains of *D. eucommiigena*, forming a well-supported monophyletic clade with 100% MLBS and 1.00 BYPP support. Another two isolates obtained in this study (SICAUCC 25-0133 and SICAUCC 25-0134) are grouped within the Section Eres, forming a distinct clade with *D. litseae*, supported by 100% MLBS and 1.00 BYPP. The network relationships of closely-related species of *D. phellodendri* and *D. leshanensis* are depicted in Fig. 2, indicating no significant recombination, based on the PHI test (*Фw* = 1).

### ﻿Taxonomy

#### ﻿ Diaporthe eucommiigena

Taxon classificationFungiDiaporthalesDiaporthaceae

S.Y. Wang, Yong Wang bis & Y. Li, Journal of Fungi 8: 1301 (2022)

2A36307B-E03D-5416-B9F0-5415F8191713

Fig. 3

##### Description.

***Saprobic*** on decaying branch of *Phellodendron chinense*. ***Sexual morph***: Not observed. ***Asexual morph***: Coelomycetous. ***Conidiomata*** 164–246 × 121–151 μm (x̄ = 208 × 132 μm, n = 10), initially immersed, becoming erumpent at maturity, discoid to conical, brown, unilocular or multilocular, mostly single-loculate, solitary or aggregated, with a distinct ostiole. ***Conidiomatal wall*** 9–24 μm wide (x̄ = 14.6 μm, n = 20), composed of multiple layers of thick-walled, pale to medium brown cells of ***textura angularis*** or ***textura globulosa***, with pigmentation gradually fading towards the interior. ***Conidiophores*** reduced to conidiogenous cells. ***Conidiogenous cells*** 5–13 × 1.4–3.8 μm (x̄ = 10.1 × 2.2 μm, n = 20), hyaline, cylindrical or pyriform, apex slightly tapering, terminal. ***Alpha-conidia*** 7.2–8.9 × 1.9–3.3 μm (x̄ = 7.9 × 2.7 μm, n = 30), hyaline, aseptate, smooth-walled, ellipsoid to fusiform, usually containing two guttules. ***Beta-conidia*** and ***Gamma-conidia*** not observed.

##### Culture characteristics.

Alpha-conidia germinated in sterile water within 24 h at 25 °C. Colonies on PDA attaining 50–55 mm diam. in 7 d at 25 °C. Colony irregular, density moderate to high, margin undulate, surface densely cottony to floccose and strongly rugose, white to creamy-white, consistency soft, elevation low-convex centrally, plane to slightly raised towards the periphery, edge well-defined. Zonation indistinct. Pycnidia scattered, more frequent near the periphery. Reverse olivaceous to dark olivaceous-brown with uneven pigmentation forming an interlaced pattern. Sporulation observed after approximately 20 d in culture, producing alpha-conidia.

**Figure 3.**
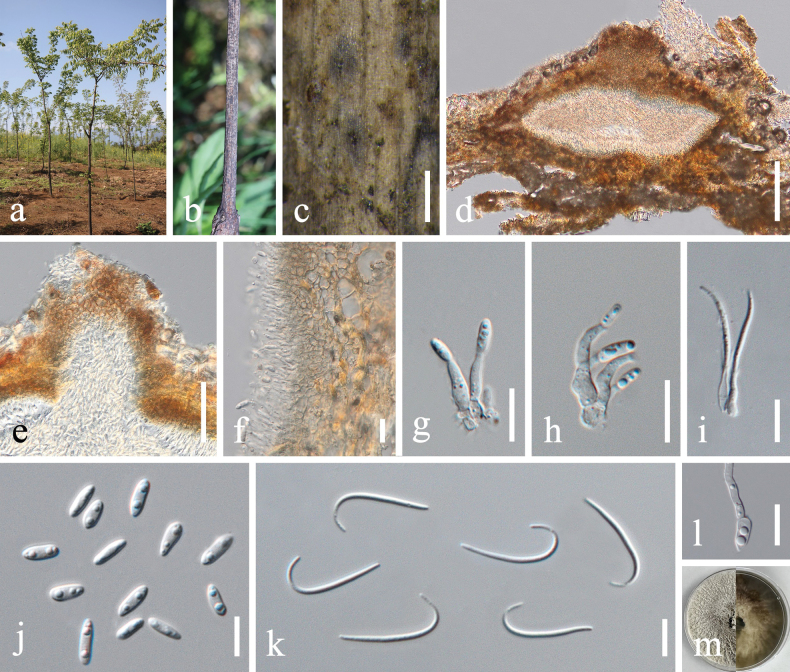
*Diaporthe eucommiigena* (SICAU 25-0159). a. Habitat of *Phellodendron chinense*; b, c. Conidiomata on host surface; d, e. Section through conidiomata; f. Ostiole; g. Conidiomatal wall; h–j. Conidiogenous cells bearing conidia; k. Alpha-conidia; l. Germinating alpha-conidium; m. Colony on PDA (left: above, right: reverse). Scale bars: 400 μm (c), 50 μm (d–f), 20 μm (g), 10 μm (h–l).

##### Material examined.

• China, Sichuan Province, Leshan City, Jinkouhe County, Jixing Village, 29°16'58.10"N, 103°13'7.42"E, 1599 m elev., from branches of *Phellodendron chinense*, 1 May 2024, X.Y. Li, LXY202405014A (SICAU 25-0159), culture (SICAUCC 25-0131); *ibid.*, LXY202405014B (SICAU 25-0160), culture (SICAUCC 25-0132).

##### Notes.

*Diaporthe eucommiigena* was originally described by Wang et al. (2022) from dead wood of *Eucommia ulmoides* in China. In a recent taxonomic revision, Dissanayake et al. (2024) treated *D. eucommiigena* as a synonym of *D. passiflorae*, based on overlapping micromorphological features of alpha conidia. However, our study revealed that these two taxa occupy distinct lineages in the multigene phylogenetic tree. Moreover, clear morphological and molecular differences support their recognition as separate species. Morphologically, beta conidia of *D. eucommiigena* are longer (27–37 × 1–2 μm vs. 16–18 × 1.5 μm), whereas its gamma conidia are smaller (7.5–10 × 1.5–2.5 μm vs. 10–12 × 2–2.5 μm) (Crous et al. 2012; Wang et al. 2022). Pairwise nucleotide comparisons further distinguish the two species. *Diaporthe passiflorae* (CBS 132527, holotype) and *D. eucommiigena* (GUCC 420.9, holotype) differ by 1.88% (11/585, 0 gap) in ITS, 6.65% (23/346, 8 gaps) in *tef*1-α and 3.04% (13/427, 1 gap) in *tub*2. Taken together, we propose the retention of *D. eucommiigena* as a distinct species, separate from *D. passiflorae*.

Two newly-obtained isolates, SICAUCC 25-0131 and SICAUCC 25-0132, clustered with *Diaporthe eucommiigena* with a strong support (100% MLBS / 1.00 BYPP; Fig. 1). Morphologically, these isolates are consistent with *D. eucommiigena*, producing cylindrical, slightly tapering conidiogenous cells and hyaline, ellipsoid to fusiform, guttulate alpha conidia (7.2–8.9 × 1.9–3.3 μm vs. 5.5–8 × 1.5–3 μm). Nucleotide comparisons between SICAUCC 25-0131 and the ex-type strain GUCC 420.9 revealed minimal variation, with 0.61% (3/488, 0 gap) in ITS, 1.11% (5/450, 1 gap) in *tub*2 and 2.82% (8/284, 6 gaps) in *tef*1-α (Table 3). Although the *tef*1-α region exhibits comparatively higher divergence, the isolates are morphologically concordant with the ex-type and differ only marginally in ITS and *tub*2 regions. Consistent with current guidance, which requires that new species be diagnosable by a unique combination of characters with at least two to three phenotypic differences (Jeewon and Hyde 2016) and recommends that slight variation in short DNA fragments should not on its own warrant taxonomic novelty, but be evaluated within an integrative framework that weighs morphology together with multilocus evidence (Jeewon and Hyde 2016; Aime et al. 2021; Chethana et al. 2021), we consider the single-locus signal from *tef*1-α insufficient to justify a new species and, therefore, retain SICAUCC 25-0131 and SICAUCC 25-0132 within *D. eucommiigena*, which is the first report on *Phellodendron chinense* in Sichuan, China.

**Table 3.** Nucleotide differences between *Diaporthe eucommiigena* (GUCC 420.9^T^) and related species.

Compared strain	Nucleotide differences		
	ITS	tef1-α	tub2
*Diaporthe passiflorae* (CBS 132527 ^T^)	1.88% (11/585 bp, 0 gap)	6.65% (23/346 bp, 8 gaps)	3.04% (13/427 bp, 1 gap)
*Diaporthe eucommiigena* (SICAUCC 25-0131)	0.61% (3/488, 0 gap)	2.82% (8/284, 6 gaps)	1.11% (5/450, 1 gap)
*Diaporthe leshanensis* (SICAUCC 25-0130 ^T^)	2.39% (12/482 bp, 1 gap)	21.77% (54/248 bp, 30 gaps)	3.11% (14/450 bp, 1 gap)
*Diaporthe phellodendri* (SICAUCC 23-0174 ^T^)	2.26% (13/574 bp, 1 gap)	20.58% (50/243 bp, 8 gaps)	3.06% (14/457 bp, 1 gap)

Note: Ex-type or ex-epitype strains are marked with “T”.

#### ﻿ Diaporthe leshanensis

Taxon classificationFungiDiaporthalesDiaporthaceae

X.Y. Li & C.L. Yang sp. nov.

59F41255-7381-55D9-B1F1-AE07C3B60554

Index Fungorum: IF903859

Fig. 4

##### Etymology.

Referring to the collection site: Leshan City in Sichuan Province, China.

##### Description.

***Saprobic*** on decaying branch of *Phellodendron chinense*. ***Sexual morph***: Not observed. ***Asexual morph***: Coelomycetous. ***Conidiomata*** 323–409 × 231–254 μm (x̄ = 370 × 244 μm, n = 10), immersed in bark, scattered, erumpent, discoid, with a solitary undivided locule. ***Conidiomatal wall*** 11–23 μm wide, parenchymatous, consisting of multi-layered pale brown, thick-walled cells of ***textura angularis*** or ***textura globulosa***. ***Conidiophores*** reduced to conidiogenous cells. ***Conidiogenous cells*** 6.7–12.3 × 1.2–3.2 μm (x̄ = 8.7 × 2.3 μm, n = 20) for producing alpha-conidia, 7.1–11.2 × 1.2–2.2 μm (x̄ = 9 × 1.6 μm, n = 20) for producing beta-conidia, terminal, enteroblastic, cylindrical, slightly tapering towards the apex. ***Alpha-conidia*** 6.6–10 × 2.1–3.2 μm (x̄ = 8.3 × 2.6 μm, n = 50), hyaline, straight, ovate to ellipsoidal, aseptate, thin-walled, base sub-truncate, usually with 2–5 guttules. ***Beta-conidia*** 19.7–22.5 × 1.3–1.7 μm (x̄ = 21.1 × 1.5 μm, n = 50), hyaline, aseptate, filiform, curved, tapering towards both ends. ***Gamma-conidia*** not observed.

##### Culture characteristics.

Alpha-conidia germinated in sterile water within 24 h at 25 °C. Colonies on PDA reached 40–50 mm in diameter after 7 d at 25 °C, with abundant, irregularly distributed, flocculent aerial mycelium. Colony surface grey-green, becoming darker with age, texture cottony to floccose, margin irregular and moderately lobate, elevation low to moderately raised. Colony density moderate to high, with sporulation observed after 30 d, producing abundant alpha-conidia. Reverse dark brown to light brown with uneven pigmentation forming an interlaced pattern.

**Figure 4.**
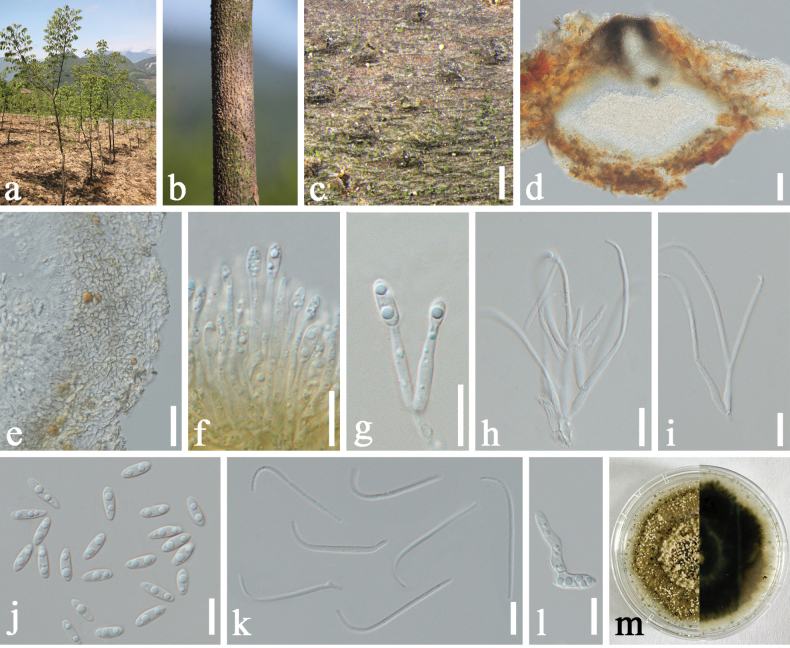
*Diaporthe leshanensis* (SICAU 25-0158, holotype). a. Habitat of *Phellodendron chinense*; b, c. Conidiomata on host surface; d. Section through a conidioma; e. Ostiole; f. Conidiomatal wall; g–i. Conidiogenous cells bearing conidia; j. Alpha-conidia; k. Beta-conidia; l. Germinating alpha-conidium; m. Colony on PDA (left: above, right: reverse). Scale bars: 500 μm (c), 150 μm (d), 50 μm (e), 10 μm (f–l).

##### Material examined.

• China, Sichuan Province, Leshan City, Jinkouhe County, Jixing Village, 29°16'58.10"N, 103°13'7.42"E, 1599 m elev., from branches of *Phellodendron chinense*, 1 May 2024, X.Y. Li, LXY202405010A (SICAU 25-0158, holotype), ex-type culture (SICAUCC 25-0130). *ibid.*, LXY202405010B (SICAU 25-0157), culture (SICAUCC 25-0129).

##### Notes.

Phylogenetic analysis of combined ITS, *tef*1-α, *tub*2, *cal* and *his*3 sequence data revealed that *Diaporthe leshanensis* formed a separate clade sister to *D. phellodendri* (100% MLBS / 1.00 BYPP; Fig. 1) and nested with *D. eucommiigena* and *D. passiflorae*. Morphologically, conidiogenous cells of *D. leshanensis* are shorter than those of *D. phellodendri* (14.1–30.4 × 1.4–2.8 μm vs. 7.1–11.2 × 1.2–2.2 μm for producing beta-conidia). Compared to *D. passiflorae*, *D. leshanensis* possesses larger alpha-conidia (6.6–10 × 2.1–3.2 μm vs. 5.5–7 × 2–3 μm) and beta-conidia (19.7–22.5 × 1.3–1.7 μm vs. 16–18 × 1.5 μm) (Crous et al. 2012). In addition, the beta-conidia of *D. leshanensis* are smaller than those of *D. eucommiigena* (19.7–22.5 × 1.3–1.7 μm vs. 27–37 × 1–2 μm) (Wang et al. 2022). Pairwise nucleotide comparisons further support the distinction of *D. leshanensis* from related taxa. *Diaporthe leshanensis* (SICAUCC 25-0130, holotype) differs from *D. phellodendri* (SICAUCC 25-0174, holotype) by 0.21% (1/482, 0 gap) differences in ITS, 3.85% (8/208, 2 gaps) differences in *tef*1-α, 1.22% (5/410, 0 gap) differences in *tub*2 and 34.6% differences (127/367, 2 gaps) in *his*3. Furthermore, sequence data between *D. leshanensis* and *D. passiflorae* (CBS 132527, holotype) showed 3.11% (15/482, 1 gap), 23.63% (60/254, 36 gaps), 2.48% (10/404, 0 gap), 5.02% (16/319, 0 gap) and 34.19% (133/389, 9 gaps) differences in ITS, *tef*1-α, *tub*2, *cal* and *his*3, respectively. Additional comparisons showed that *D. leshanensis* differs from *D. eucommiigena* (GUCC 420.9) by 2.39% (12/482, 1 gap) in ITS, 21.77% (54/248, 30 gaps) in *tef*1-α and 3.11% (14/450, 1 gap) in *tub*2. Hence, based on its morphological characteristics, phylogenetic analysis, and nucleotide polymorphism comparison, *D. leshanensis* is described here as a new species.

#### ﻿ Diaporthe litsease

Taxon classificationFungiDiaporthalesDiaporthaceae

Y.R. Sun, Yong Wang bis & K.D. Hyde, Phytotaxa 665(3): 248 (2024)

F53BF97A-49BE-5158-9F95-E282976220B2

Fig. 5

##### Description.

***Saprobic*** on decaying branch of *Phellodendron chinense*. ***Sexual morph***: Not observed. ***Asexual morph***: Coelomycetous. ***Conidiomata*** 298–341 × 207–257 μm (x̄ = 316 × 228 μm, n = 10), solitary, scattered, dark brown to black, discoid to elliptical, unilocular. ***Conidiomatal wall*** 13–48 μm wide, parenchymatous consisting of multi-layers of brown, thick-walled cells of ***textura angularis*** or ***textura globulosa***. ***Conidiophores*** hyaline, smooth, straight to flexural, basally branched or unbranched. ***Conidiogenous cells*** 12–23 × 1.7–2.8 μm (x̄ = 16 × 2.3 μm, n = 20) for producing alpha-conidia, 11–15 × 1.2–2.5 μm (x̄ = 12.6 × 1.7 μm, n = 20) for producing beta-conidia, terminal, enteroblastic, monophialidic, cylindrical, slightly tapering towards the apex. ***Alpha-conidia*** 6–10 × 2.3–3.3 μm (x̄ = 8.4 × 2.7 μm, n = 50), hyaline, aseptate, ellipsoid to fusiform, smooth-walled, containing 1–5 guttules, base subtruncate. ***Beta-conidia*** 27–36 × 1.1–1.8 μm (x̄ = 32 × 1.4 μm, n = 50), hyaline, aseptate, filiform, curved, tapering towards both ends. ***Gamma-conidia*** not observed.

##### Culture characteristics.

Alpha-conidia germinated in sterile water within 24 h at 25 °C. Colonies on PDA reached 55–60 mm in diameter after 7 d at 25 °C. Colony circular to slightly lobate, distinctly zonate, with a darker olivaceous-brown central disc surrounded by a thick pale buff to grey-green annulus of floccose tufts and an outer olivaceous ring. Surface uneven, numerous punctiform pycnidia, densest in the central disc. Aerial mycelium dense and cottony-floccose, forming small fasciculate tufts, margin entire to slightly lobate, with a thin band of submerged hyaline mycelium at the extreme edge. Reverse blackish to dark brown in the centre, becoming olivaceous-brown and then light brown towards the margin, with a conspicuous diffusing olivaceous-brown pigment in the PDA. Sporulation observed after approximately 20 d in culture, producing abundant alpha-conidia.

**Figure 5.**
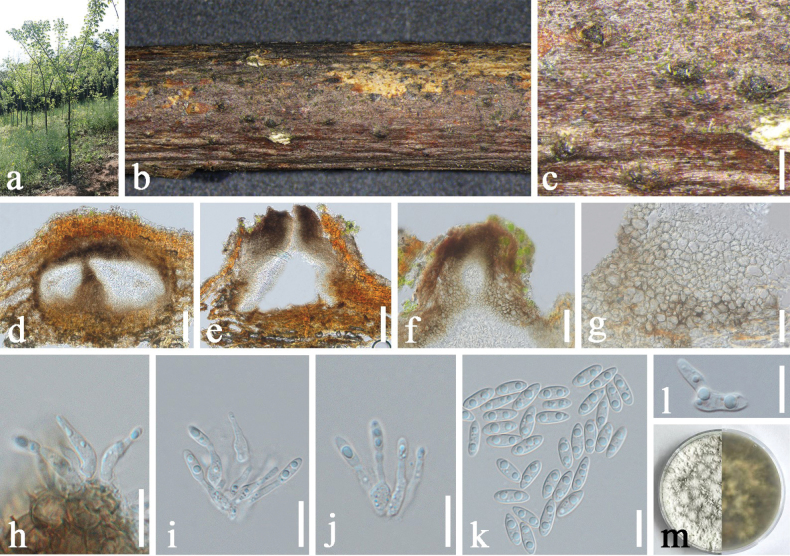
*Diaporthe litsease* (SICAU 25-0161). a. Habitat of *Phellodendron chinense*; b, c. Conidiomata on host surface; d. Section through a conidioma; e. Conidiomatal wall; f–i. Conidiogenous cells bearing conidia; j. Alpha-conidia; k. Beta-conidia; l. Germinating alpha-conidium; m. Colony on PDA (left: above, right: reverse). Scale bars: 500 μm (c), 50 μm (d, e), 10 μm (f–l).

##### Material examined.

• China, Sichuan Province, Leshan City, Jinkouhe County, Jixing Village, 29°16'58.10"N, 103°13'7.42"E, 1599 m elev., from branches of *Phellodendron chinense*, 1 May 2024, X.Y. Li, LXY202405031A (SICAU 25-0161), culture (SICAUCC 25-0133); *ibid.*, LXY202405031B (SICAU 25-0162), culture (SICAUCC 25-0134).

##### Notes.

*Diaporthe litseae* was described as an endophyte on healthy leaves of *Litsea kobuskiana* from Guizhou Province, China (Sun et al. 2024). In our phylogenetic analysis, two isolates obtained from branches of *Phellodendron chinense* grouped within the *D. litseae* clade with strong statistical support (100% MLBS / 1.00 BYPP; Fig. 1). Morphologically, the new isolates exhibit characteristics consistent with *D. litseae*, particularly in having ellipsoid to fusiform, multiguttulate alpha conidia (6–10 × 2.3–3.3 μm vs. 5–9 × 2–3.5 μm). Pairwise sequence comparisons between our representative strain SICAUCC 25-0133 and the ex-type strain of *D. litseae* (GUCC 23-0055) revealed high similarity, viz. 99.61% in ITS (508/510 bp, 1 gap), 97.98% in *tef*1-α (291/297 bp, 0 gap), 100% in *his*3 (422/422 bp, 0 gap) and 99.33% in *cal* (446/449 bp, 0 gap). Therefore, we identify the new collections as *D. litseae*, marking the first record on *P. chinense* as saprobe in Sichuan, China.

#### ﻿ Diaporthe phellodendri

Taxon classificationFungiDiaporthalesDiaporthaceae

X.Y. Li & C.L. Yang sp. nov.

3258F21B-17F5-5F3D-8DA8-C43A9341BBAE

Index Fungorum: IF901258

Fig. 6

##### Etymology.

Refers to the host genus *Phellodendron*.

##### Description.

***Saprobic*** on decaying branch of *Phellodendron chinense*. ***Sexual morph***: Not observed. ***Asexual morph***: Coelomycetous. ***Conidiomata*** 257–291 × 211–240 μm (x̄ = 271 × 223 μm, n = 10), immersed, discoid to conical or irregular, brown to dark brown, solitary, scattered, unilocular. ***Conidiomatal wall*** 17–40 μm wide, parenchymatous consisting of multi-layers of pale brown to reddish-brown, thick-walled cells of ***textura angularis*** or ***textura globulosa***. ***Conidiophores*** hyaline, smooth, straight to flexural, basally branched or unbranched. ***Conidiogenous cells*** 6.7–37 × 1.2–2.7 μm (x̄ = 18.7 × 1.8 μm, n = 20) for producing alpha-conidia, 14.1–30.4 × 1.4–2.8 μm (x̄ = 22.1 × 1.9 μm, n = 20) for producing beta-conidia, terminal, enteroblastic, monophialidic, cylindrical, slightly tapering towards the apex. ***Alpha-conidia*** 6.6–9.7 × 1.9–3.2 μm (x̄ = 8.1 × 2.6 μm, n = 50), hyaline, straight, ovate to ellipsoidal, aseptate, thin-walled, base sub-truncate, usually with two guttules. ***Beta-conidia*** 16.3–27.1 × 1.5–1.7 μm (x̄ = 20.4 × 1.6 μm, n = 50), hyaline, aseptate, filiform, curved, tapering towards both ends, scattered amongst the alpha conidia. ***Gamma-conidia*** not observed.

##### Culture characteristics.

Alpha-conidia germinated in sterile water within 24 h at 25 °C. Colonies on PDA attaining 40–50 mm diam. in 7 d. Colony irregular, with a fimbriate margin, mycelium sparse, surface floccose to cottony. Initially producing white aerial mycelium appressed to the medium surface, later developing into off-white to greyish-white colonies. Colony slightly raised, highest at the centre and gradually lower towards the margin. Reverse pale yellow with uneven pigmentation. Sporulation observed after approximately 20 d in culture, forming pale yellow to brown conidial masses irregularly distributed across the colony.

**Figure 6.**
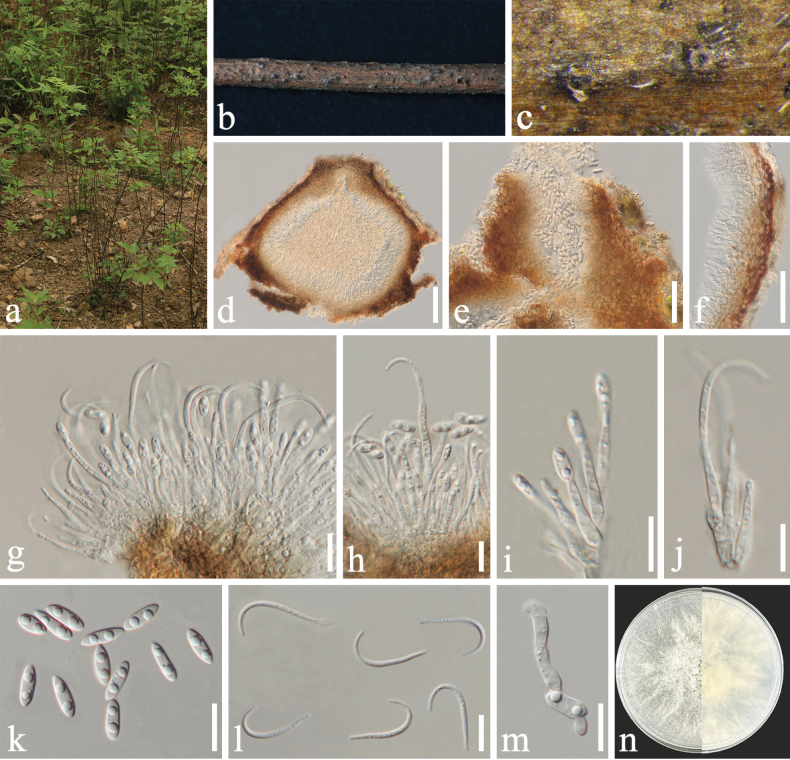
*Diaporthe phellodendri* (SICAU 24-0065, holotype). a. Habitat of *Phellodendron chinense*; b, c. Conidiomata on host surface; d. Section through a conidioma; e. Ostiole; f. Conidiomatal wall; g–j. Conidiogenous cells bearing conidia; k. Alpha-conidia; l. Beta-conidia; m. Germinating alpha-conidium; n. Colony on PDA (left: above, right: reverse). Scale bars: 200 μm (c), 50 μm (d), 30 μm (e, f), 10 μm (g–m).

##### Material examined.

• China, Sichuan Province, Yibin City, Junlian County, Haoba Village, 27°55'20.16"N, 104°33'23.89"E, 1337 m elev., from branches of *Phellodendron chinense*, 2 May 2024, X.Y. Li, LXY202405030A (SICAU 24-0065, holotype), ex-type culture (SICAUCC 23-0174); *ibid.*, LXY202405030B (SICAU 24-0066), culture (SICAUCC 23-0175).

##### Notes.

In the phylogenetic analysis, *Diaporthe phellodendri* formed a well-supported clade (100% MLBS / 1.00 BYPP; Fig. 1), showing its closest affinities to *D. eucommiigena*, *D. passiflorae* and *D. leshanensis*. Morphologically, *D. phellodendri* differs from *D. passiflorae* by having larger alpha conidia (6.6–9.7 × 1.9–3.2 μm vs. 5.5–7 × 2–3 μm), as well as larger beta conidia (16.3–27.1 × 1.5–1.7 μm vs. 16–18 × 1.5–2 μm) (Crous et al. 2012). In contrast to *D. eucommiigena*, *D. phellodendri* produces smaller beta conidia (16.3–27.1 × 1.5–1.7 μm vs. 27–37 × 1–2 μm) (Wang et al. 2022). Pairwise nucleotide comparisons further support the distinctiveness of *D. phellodendri*. It differs from *D. passiflorae* (CBS 132527, holotype) by 2.78% in ITS (16/575 bp, 1 gap), 19.57% in *tef*1-α (45/230 bp, 8 gaps), 3.75% in *tub*2 (16/427 bp, 0 gap) and 7.69% in *his*3 (31/403 bp, 6 gaps). In comparison with *D. eucommiigena* (GUCC 420.9, holotype), *D. phellodendri* shows sequence divergence of 2.26% in ITS (13/574 bp, 1 gap), 20.58% in *tef*1-α (50/243 bp, 8 gaps) and 3.06% in *tub*2 (14/457 bp, 1 gap). In addition, *D. phellodendri* is clearly separable from *D. leshanensis*, with diagnostic characters provided in the description of the latter. Therefore, *D. phellodendri* is described as a new species within Section Sojae.

## ﻿Discussion

According to the United States Department of Agriculture (USDA) Fungal Database (https://fungi.ars.usda.gov/, accessed 20 May 2025), only five fungal species have been previously reported on *Phellodendron chinense*, including *Coleosporium phellodendri*, *Diplodia rutaecola*, *Heteroconium phellodendri*, *Nigrospora guilinensis* and *Passalora phellodendricola* (Zhang et al. 1997; Crous and Braun 2003; Dudka et al. 2004; Ma et al. 2012; Zeng et al. 2020). To date, the occurrence of *Diaporthe* species on this host has not been documented. In the present study, eight *Diaporthe* isolates were obtained from decaying branches of *Phellodendron chinense*. Phylogenetic analyses, based on a five-locus dataset (ITS, *tef*1-α, *tub*2, *cal* and *his*3) combined with morphology, supported the introduction of two new species, *Diaporthe phellodendri* and *D. leshanensis*. In addition, *D. eucommiigena* and *D. litseae* were isolated from *P. chinense* for the first time. These findings expand the known host range of *Diaporthe* and refine species delimitation within the genus.

The taxonomic delimitation of *Diaporthe* species continues to present significant challenges, particularly given the dynamic re-definition of sectional classifications (Hilário et al. 2021a, b; Norphanphoun et al. 2022; Pereira et al. 2023; Dissanayake et al. 2024; Ferro et al. 2024; Li et al. 2025; Zhang et al. 2025). Recent systematic revisions have delineated seven sections within the genus: Betulicola, Crotalariae, Eres, Foeniculina, Psoraleae-pinnatae, Rudis and Sojae (Dissanayake et al. 2024). Our phylogenetic analyses placed three species (*Diaporthe eucommiigena*, *D. phellodendri* and *D. leshanensis*) within Section Sojae, which contains nine species/species-complexes. Within the section, these taxa occupy lineages outside the currently recognised species/species-complexes and, together, form a strongly supported clade with *D. passiflorae*. Historically, *D. eucommiigena* was synonymised with *D. passiflorae* due to overlap in alpha conidial characters (Dissanayake et al. 2024). However, our multilocus phylogeny resolves them as distinct sister lineages, corroborated by differences in beta and gamma conidial dimensions and by divergence across ITS, *tef*1-α and *tub*2. Likewise, *D. litseae* is phylogenetically nested within Section Eres, which contains five species/species-complexes, but occupies an isolated position outside the established species/species-complexes. This study further expands the morphological characterisation of *D. litseae* by documenting its previously undescribed beta-conidia. This refined morphological profile facilitates a more comprehensive understanding of its morphological variability and will contribute to enhancing future identification efforts within Section Eres.

In recent years, China has become a hotspot for *Diaporthe* research, with numerous studies documenting novel taxa and expanding host records across diverse ecosystems. Based on the USDA Fungal database (https://fungi.ars.usda.gov/, accessed 15 August 2025), 389 records from China across 63 host families indicate that *Diaporthe* is concentrated in a subset of woody, economically important families, particularly Rutaceae, Rosaceae and Theaceae, with notable representation in Fagaceae, Actinidiaceae, Sapindaceae and Vitaceae. This pattern is consistent with recent studies on diverse plant hosts across China that collectively document substantial *Diaporthe* diversity and associations with important plant diseases (Huang et al. 2015; Gao et al. 2016; Dissanayake et al. 2020; Yang et al. 2020; Wang et al. 2021; Cao et al. 2022; Lu et al. 2022; Luo et al. 2022; Bai et al. 2023; Xiao et al. 2023; Liu et al. 2024; Zhu et al. 2024; Li et al. 2025; Shao et al. 2025; Zhang et al. 2025).

Sichuan Province, located at the intersection of diverse climatic and topographic zones, harbours rich biodiversity of medicinal plants and provides unique ecological niches for fungal colonisation. Despite its status as a key region for authentic Chinese medicinal plants, fungal surveys targeting woody medicinal plants in Sichuan remain limited. The identification of *Diaporthe* taxa from *Phellodendron chinense* underscores the underexplored fungal diversity associated with woody medicinal plants in this region. Future studies should survey fungal communities on woody medicinal hosts, focusing on their pathogenic potential to clarify disease patterns and inform sustainable management of forest-derived medicinal resources in this ecologically rich region.

## Supplementary Material

XML Treatment for Diaporthe eucommiigena

XML Treatment for Diaporthe leshanensis

XML Treatment for Diaporthe litsease

XML Treatment for Diaporthe phellodendri

## ﻿Additional information

### Use of AI

No use of AI was reported.

### Author ORCIDs

Xinyue Li https://orcid.org/0009-0006-5189-0922

Xiulan Xu https://orcid.org/0000-0002-6832-5421

Feng Liu https://orcid.org/0000-0003-4580-7169

Chunlin Yang https://orcid.org/0000-0002-3854-8835

### Data availability

All of the data that support the findings of this study are available in the main text.
